# When Screens Replace Sleep: Short Video Addiction Impairs Executive Function Through Sleep Disruption in Youth—Is Physical Activity the Antidote?

**DOI:** 10.1002/brb3.71369

**Published:** 2026-04-16

**Authors:** Ting Xiao, Haiou Peng, Zhiru Liang, Mengting Pan, Yang Liu, Yanfei Wang, Bo Wang, Wenxi Tang

**Affiliations:** ^1^ Beijing Sport University Beijing China; ^2^ Hunan University of Medicine Huaihua China; ^3^ Department of Hepatobiliary Surgery Xilingol League Central Hospital Xilinhot Inner Mongolia China; ^4^ Southwest University Chongqing China; ^5^ Jishou University Jishou China; ^6^ Department of Science and Education Xilingol League Central Hospital Xilinhot Inner Mongolia China; ^7^ Baotou Medical College Inner Mongolia University of Science and Technology Baotou Inner Mongolia China

**Keywords:** Adolescents, Executive function, Physical activity, Short Video Addiction, Sleep quality

## Abstract

**Background:**

This study investigated the association between short video addiction (SVA) and executive function (EF) in adolescents, incorporating sleep quality (SQ) as a mediator and physical activity (PA) as a moderator.

**Methods:**

A sample of 978 adolescents from Hunan and Sichuan provinces was assessed using standardized measures of SVA, EF, PA, and SQ.

**Results:**

Significant demographic differences were observed, with males reporting lower SVA and higher PA than females, while only children, urban residents, and non‐left‐behind adolescents showed more favorable outcomes across key variables. Correlation analyses revealed that SVA was negatively associated with SQ, PA, and EF, whereas SQ, PA, and EF were mutually positively correlated. Mediation analysis identified SQ as a significant partial mediator in the SVA–EF relationship, accounting for 8.11% of the total effect. Moderated mediation analysis showed that PA significantly moderated the direct pathway between SVA and EF: as PA levels increased, the negative association between SVA and EF became more pronounced, whereas PA did not exert a regulatory influence on the SVA–SQ pathway.

**Conclusions:**

These findings suggest that while SQ partially mediates the SVA–EF relationship, PA serves as a significant moderator in the direct pathway, with important implications for addressing adolescent SVA and supporting EF development through sleep quality enhancement and physical activity promotion.

## Graphical abstract

Using a sample of 978 Chinese adolescents, this graphical abstract presents a moderated mediation model in which sleep quality (SQ) partially mediates the negative effect of short video addiction (SVA) on executive function (EF), while physical activity (PA) moderates the direct SVAEF pathway. Path coefficients indicate that higher PA exacerbates the negative direct effect of SVA on EF, whereas PA does not moderate the SVASQ link. The findings highlight that promoting physical activity and sleep quality while reducing addiction may collectively support executive function in adolescents.

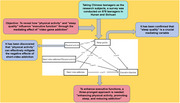

## 1 Introduction

Under the driving force of artificial intelligence and 5G technology (Yang et al. 2023), short videos have evolved into a mainstream medium due to their fragmented dissemination, strong interactivity, and immersive experience (Chung et al. 2021). There is a growing recognition that compulsive consumption of this media carries a propensity for addiction, marking it as a nascent form of social risk (Zhang et al. 2024). According to the “Blue Book on Teenagers: Annual Report on Internet Use by Chinese Minors (2021),” adolescents (who account for 49.3% of underage internet users) (Lu et al. 2022) have become the core group of users, with their behaviors mainly concentrated on platforms such as Douyin (60.4%) and other platforms (59.3%) (Zhao and Kou 2023). As of June 2023, short video platforms had achieved a penetration rate of 95.2% among Chinese internet users, with the total user count surpassing the one billion mark. Emerging as a widespread concern, Short Video Addiction (SVA) represents a specific form of social media addiction and is increasingly recognized as a distinct category within internet addiction disorders(Ye et al. 2022; Zhang et al. 2019). This addiction is induced through high‐density information flows lasting 1 to 5 min (Zhang et al. 2019) and emotional compensation mechanisms (such as nostalgia fulfillment and emotional escape) (Liu and Huang et al., 2025), leading to behavioral dysregulation (Ye et al. 2022). It has been shown to result in a 17.2% decrease in learning efficiency (Qin Yao) and is significantly associated with anxiety and depression (Haoxuan et al. 2019), sleep disorders (Zhang et al. 2022), memory decline (Sha and Dong 2021), and other related health issues. Further research reveals that addiction impairs the function of the prefrontal cortex (Pryde and Prichard 2022), a region responsible for executive control functions, and its damage exacerbates behavioral regulation deficits.

Studies indicate that individuals with internet addiction frequently demonstrate impairments in executive function, with such deficits becoming increasingly prevalent among this population (Rosselli and Christopher, 2019). Executive function (EF) refers to a set of higher‐order cognitive processes necessary for situations requiring focused attention and sustained concentration, particularly when automatic responses, instincts, or intuitive actions are inadequate or inappropriate (Burgess and Simons 2005; Espy 2004). Serving as the fundamental mechanism governing behavior regulation, EF encompasses capabilities such as cognitive flexibility, monitoring of working memory, and control over impulses (Goldberg and Verdejo‐Garcia 2017). Within the framework of cognitive neuroscience, EF can be adversely affected by patterns of addiction and substance use disorders, which may induce enduring alterations in neural circuitry and disrupt prefrontal cortex functionality (van der Niet et al. 2015), especially within descending regulatory systems (Bates et al. 2006). Internet addiction not only compromises inhibitory control but is also linked to more extensive deficiencies in global executive processes, including decision‐making and working memory (Ioannidis et al. 2019).

Sleep quality (SQ) involves key dimensions such as sleep onset latency, sleep continuity, and subjective recovery (Shin et al. 2013). Exposure to both endogenous and exogenous influences over extended periods may contribute to the development of sleep disturbances (J. Peng, M. Liu, Z. Wang, and L. Xiang et al. 2025; J. Peng and J. Wang et al. 2025; Xiao et al. 2024), such as insomnia, hypersomnia, and other disorders of sleep (Spytska 2024). Insomnia, as a typical issue of poor SQ, significantly contributes to reduced alertness, distracted attention, and impaired memory function (Shekleton et al. 2014). Emerging evidence indicates that compulsive short‐form video consumption, fueled by the growth of these platforms, is now recognized as a key determinant of poor SQ (Zhang et al. 2017), raising concerns about its usage behavior. Sleep issues have been consistently associated with SVA in empirical research (Alonzo et al. 2021; Levenson et al. 2016): using short videos before sleep prolongs sleep onset latency and increases the frequency of nighttime awakenings (Thomée et al. 2010). This effect may be related to the suppression of melatonin secretion by smartphone screen light and cognitive arousal. Sleep plays a crucial role in brain metabolism and the clearance of waste products, as well as in neural plasticity (Albrecht and Ripperger 2018). Chronic poor SQ exacerbates EF deficits, and cognitive decline further worsens sleep, creating a vicious cycle (Cricco et al. 2001). Due to the long‐term negative interactions between sleep disorders and EF impairments, improving SQ may enhance cognitive abilities or delay cognitive decline. Relevant research indicates that with improved SQ, the decline in many cognitive abilities can be reversed (Waters and Bucks 2011).

The multidimensional impact of SVA has attracted scholarly attention, particularly regarding the potential of physical activity (PA) as a non‐pharmacological intervention. Research indicates that regular PA not only improves internet addiction (Gan et al. 2025; Xiao et al. 2024; Yin et al. 2025), mobile phone dependency (Pirwani and Szabo 2024), and substance addiction (Wang et al. 2014) through the regulation of neurotransmitter levels (such as the homeostasis of the dopamine system), but also holds alleviative potential for SVA (Jianfeng et al. 2024; Luo et al. 2023; Zhang et al. 2022). The mechanisms underlying this effect involve multiple pathways: moderate‐to‐high intensity aerobic exercise enhances neural plasticity in the prefrontal cortex (Colcombe and Kramer 2003), breaking the cognitive dysregulation cycle associated with addictive behaviors by improving EF. Additionally, PA modulates the expression of core genes related to circadian rhythms (Passos et al. 2012), thereby improving sleep disturbances induced by short video use before bedtime (Luo et al. 2023). The positive impact of physical activity PA on mental health is well‐established (Liu et al. 2024), with empirical studies confirming its protective effect against internet addiction and other dependency‐related behaviors (Jia et al. 2025; Liu et al. 2024; J. Peng, Y. Liu, X. Wang, and Z. Yi et al. 2025). The pleasure derived from short video consumption is central to the addictive experience, with flow theory positing that individuals experience intense pleasure during deep focus and active engagement. PA can replace this flow state, fulfilling psychological needs and reducing reliance on short videos, especially when individuals are fully immersed in the activity, triggering dopamine release—thereby enhancing the effect of reducing dependence (Nakamura and Csikszentmihalyi 2009).

**FIGURE 1:**
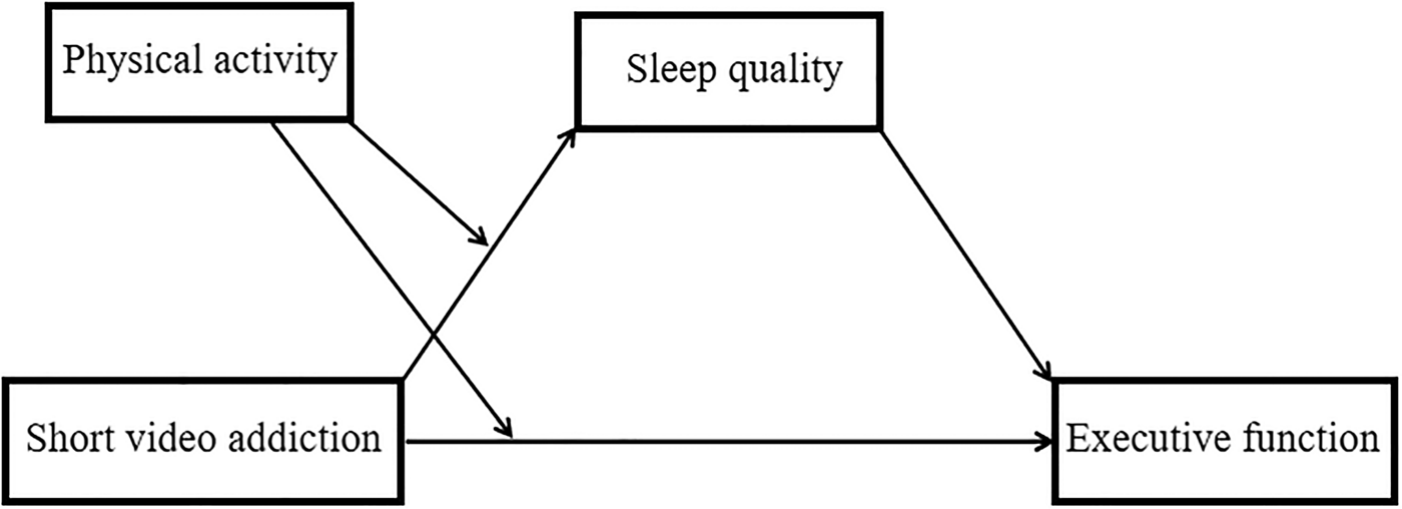
Brokerage mediation model diagram.

Based on the theoretical analysis of the relationships among SVA, EF, SQ, and PA in the existing literature, the present study aims to explore the impact of SVA on EF, while also exploring the mediating role of SQ and the moderating influence of PA. These relationships were analyzed using a moderated mediation model (Figure 1), which outlines the pathways linking adolescent SVA to EF. Within this framework, SQ reflects the underlying psychological mechanism, and PA acts as a contextual moderator. Accordingly, the following hypotheses were formulated:

1. H1: Adolescent SVA is significantly negatively correlated with EF.
2. H2: SQ mediates the association between SVA and EF among adolescents.
3. H3: PA significantly moderates the relationship between SVA and EF.
4. H4: PA significantly moderates the relationship between SVA and SQ.

## 2 Methods

### 2.1 Participants

The data collection for this cross‐sectional survey was carried out in November 2025. The research subjects were middle school students from Hunan and Sichuan provinces. Participants were eligible for inclusion if they were aged 10–18 years and voluntarily agreed to participate and could independently complete the questionnaires.Responses were excluded if they were incomplete (with at least one unanswered item) or showed patterned inconsistent responses (e.g., selecting the same option for all items). Prior to the questionnaire distribution, we obtained written informed consent from all participants and their guardians after explaining the purpose, procedures, data confidentiality, and potential risks of the study in detail. Guardians of minor participants were informed of the study content through parent‐teacher meetings and signed the consent form voluntarily. The survey was conducted anonymously on a voluntary basis, with an anticipated duration of 20 min for completion. The study protocol received prior approval from the Biomedical Ethics Committee at the authors’ affiliated institution. All investigative procedures conformed to the ethical standards outlined in the Declaration of Helsinki. Initially, 1098 students completed the survey. After eliminating responses that were either incomplete or showed pattern inconsistencies, the final analytic sample included 978 individuals (468 boys, 510 girls), with an average age of 14.77 years (SD = 1.58).

### 2.2 Short Video Addiction

The severity of SVA was measured using the “Short Video Addiction Scale” introduced by Mao Zheng and colleagues (2023). This instrument contains 13 items, each scored on a 5‐point Likert system from 1 (Strongly Disagree) to 5 (Strongly Agree). Cumulative scores fall between 13 and 65, where elevated totals reflect a greater degree of addictive behavior. In the current study, the measure showed high internal reliability, achieving a Cronbach's α value of 0.889.

### 2.3 Executive Function

The “Adolescent Executive Function Scale,” developed by Huang et al. (2014), was used to assess EF. This scale includes three dimensions: working memory, cognitive flexibility, and inhibitory control, with a total of 21 items. A 3‐point scoring system was applied, where 1 corresponds to “Always,” 2 to “Sometimes,” and 3 to “Never.” The total score ranges from 21 to 63, with higher scores indicating better EF. The Cronbach's α coefficient for this scale in the present study was 0.924.

### 2.4 Sleep Quality

SQ in this study was assessed using a single‐item measure (Snyder et al. 2018). This 10‐point scale is widely used in adolescent sleep research with good reliability and validity; its concise design reduces response burden while covering core dimensions of sleep quality. Participants were asked to respond to the following question: “Please reflect on the overall quality of your sleep over the past 7 days. This includes the duration of your sleep, the ease of falling asleep, the frequency of waking up at night (excluding waking due to the need to use the restroom), unnecessary early awakening, and your overall feeling of refreshment upon waking. How would you rate the quality of your sleep over the past week?” SQ was assessed via a widely adopted 10‐point scale, a measure chosen for its proven reliability. On this scale, a higher rating is indicative of better SQ (Weng et al. 2022; Yin et al. 2025).

### 2.5 Physical Activity

PA was measured with the “Physical Activity Level Scale” modified by Liang (1994). This tool consists of three domains: intensity, time, and frequency of activity. A composite score was calculated based on the equation: Intensity × (Duration − 1) × Frequency, resulting in a total ranging from 0 to 100. Higher values correspond to more frequent and intense engagement in physical activities. In the current sample, the scale exhibited satisfactory reliability, with a Cronbach's α of 0.725.

### 2.6 Covariates

In this study, gender (dichotomous variable), grade (ordinal categorical variable), residential status (urban‐rural dichotomous variable), only‐child status (dichotomous variable), and left‐behind status (dichotomous variable) were included as covariates, based on evidence from previous research: existing studies have confirmed that these demographic variables exhibit significant heterogeneity across different groups. For example, in terms of gender, indicators such as sleep quality and daily behavioral patterns among adolescents show statistical differences between males and females; variables such as grade and residential status are also associated with individual behavioral characteristics and environmental exposure levels. These confounding factors may interfere with the association analysis of the core variables in this study (Jiang and Yoo 2024; B. Peng et al. 2025; Yang and Liu 2025; Ye et al. 2025; Zhao and Kou 2023). Therefore, this study collected data on the above variables through categorical items and simultaneously incorporated these covariates into the multivariate analysis model to achieve statistical control, thereby enhancing the internal validity of the research conclusions.

### 2.7 Data Analysis

Statistical analyses were performed with SPSS 26.0, encompassing differential and correlation analyses, as well as assessments of mediation and moderation effects. An initial check for common method bias was carried out, applying a cutoff of 40% to identify substantial bias (Podsakoff et al. 2003). After confirming that no notable bias was present, descriptive statistics and correlation coefficients were generated for demographic characteristics and core variables. Key variables were standardized to facilitate further analysis. The hypothesized mediation and moderated mediation pathways were tested utilizing the PROCESS macro (Models 14 and 18) within SPSS. This approach enabled assessment of SQ as a mediator between SVA and adolescent EF, as well as examination of the direct influence of SVA on EF incorporating both the mediating role of SQ and moderating influence of PA. Model reliability was evaluated using 5,000 bootstrap samples to derive 95% confidence intervals (95% CI), enhancing the robustness of inferences. The regression procedures implemented through PROCESS employed bias‐corrected bootstrapping with 5,000 resamples (Hayes 2018). Effects were considered statistically significant if the 95% bootstrap CI excluded zero, with a significance level set at α = 0.05.

## 3 Research Results

### 3.1 Common Method Bias Test

To assess the possible impact of common method bias, Harman's single‐factor test was performed. The results indicated four factors with eigenvalues greater than 1.0 before rotation. The initial factor explained 25.51% of the total variance, remaining below the critical cutoff of 40%. Therefore, common method bias did not represent a major source of bias in this investigation.

### 3.2 Descriptive Analysis

The results presented in Table 1 reveal significant differences in SVA, PA, and EF across gender, only‐child status, living situation, left‐behind status, and grade levels. Additionally, SQ exhibited significant differences based on living situation, left‐behind status, and grade levels (see Table 2).

**TABLE 1:** Descriptive statistics of population variables.

Category	Subgroup	*N*	Percent
Gender	Boys	468	47.9%
	Girls	510	52.1%
Single child situation	Only child	215	22.0%
	non‐only child	763	78.0%
Living conditions	City	685	70.0%
	Rural area	293	30.0%
Guarding situation	Stay behind	477	48.8%
	Non‐resident	501	51.2%
Grade	Primary school	35	3.6%
	Junior high school	389	39.8%
	Senior high school	554	56.6%

**TABLE 2:** Describes the analysis.

Variables		SVA		SQ		PA		EF	
		Mean	SD	Mean	SD	Mean	SD	Mean	SD
Gender	Boys	35.38	11.22	5.57	2.42	22.20	22.14	47.87	8.68
	Girls	38.38	10.31	5.51	2.34	17.86	18.82	46.17	8.76
	*t*	−4.352***		0.399		3.288**		3.048**	
Single child situation	Only child	33.68	10.14	5.80	2.55	27.23	21.97	49.35	9.76
	Non‐only child	37.87	10.87	5.46	2.32	17.88	19.70	46.31	8.34
	*t*	−5.052***		1.859		5.634***		4.158***	
Living conditions	City	35.81	10.90	5.80	2.35	22.50	20.75	47.98	8.92
	Rural area	39.60	10.28	4.92	2.32	13.93	18.90	44.66	7.91
	*t*	−5.180***		5.390***		6.307***		5.787***	
Guarding Situation	Stay behind	38.60	10.43	4.86	2.25	14.20	16.46	44.81	8.04
	Non‐resident	35.37	11.02	6.19	2.31	25.39	22.55	49.06	8.92
	*t*	4.707***		−9.142***		−8.894***		−7.835***	
Grade	Primary school	41.60	11.86	4.54	2.42	8.17	13.62	41.74	8.59
	Junior high school	32.51	10.49	6.30	2.47	29.12	23.50	50.50	9.15
	Senior high school	39.77	9.95	5.07	2.16	14.23	15.77	44.84	7.57
	F	61.081***		36.484***		75.866***		60.894***	

**: *p*<0.01; ***: *p*<0.001.

### 3.3 Correlation Analysis

The findings indicated substantial inverse relationships between SVA and SQ (with a correlation coefficient of *r* = −0.300, *p* < 0.001), PA (*r* = −0.293, *p* < 0.001), and EF (*r* = −0.483, *p* < 0.001). Furthermore, SQ was significantly and positively associated with both PA (*r* = 0.267, *p* < 0.001) and EF (*r* = 0.312, *p* < 0.001). Additionally, a notable positive association was observed between PA and EF (*r* = 0.253, *p* < 0.001), as detailed in Table 3.

**TABLE 3:** Correlation analysis.

Variables	SVA	SQ	PA	EF
1 SVA	—			
2 SQ	−0.300***	—		
3 PA	−0.293***	0.267***	—	
4 EF	−0.483***	0.312***	0.253***	—

***: *p*<0.001.

### 3.4 Mediation Model Testing

The results indicated that SQ partially mediated the relationship between SVA and EF. Specifically, a significant negative correlation was found between SVA and SQ (β = −0.241, *t* = −7.674, *p* < 0.001), as well as between SVA and EF (direct effect: β = −0.419, *t* = −14.390, *p* < 0.001). After including SQ in the model, the relationship between SVA and EF weakened (indirect effect: β = −0.386, *t* = −12.996, *p* < 0.001), while a significant relationship between SQ and EF remained (β = 0.139, *t* = 4.719, *p* < 0.001) (see Table 4).

**TABLE 4:** Tests the mediation model.

Outcome variables	Predictor variables	β	SE	*t*	*R* ^2^	*F*
SQ	SVA	−0.241	0.032	−7.674***	0.158	30.347***
EF	SVA	−0.419	0.029	−14.390***	0.277	62.0165***
EF	SVA	−0.386	0.030	−12.996***	0.293	57.502***
	SQ	0.139	0.029	4.719***		

***: *p*<0.001.

**FIGURE 2:**
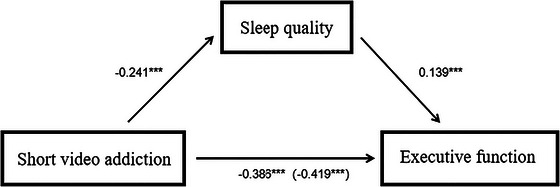
Mediating model diagram.

According to the path analysis outcomes of the mediation model depicted in the illustration, the overall impact of SVA on EF was ‐0.419, accompanied by a standard deviation of 0.029 and a 95% bootstrap CI ranging from −0.477 to −0.362. Upon accounting for SQ as a mediator, the direct influence of SVA on EF was ‐0.386, with a standard deviation of 0.030 and a 95% bootstrap CI of [−0.444, −0.328], signifying that the direct impact continued to be notable. Moreover, SVA had an indirect relationship with EF through SQ, with the indirect relationship value at −0.034, a standard deviation of 0.009, and a 95% bootstrap confidence interval of [−0.053, −0.017], which also achieved statistical significance. The indirect relationship accounted for 8.11% of the total relationship between SVA and EF, indicating that SQ exerted a partial mediating role in the relationship between SVA and EF (see Table 5 and Figure 2).

**TABLE 5:** Path analysis of mediation model.

Intermediate path	Effect size	SE	Bootstrap 95% CI	Proportion of mediating effect
Total effect	−0.419	0.029	−0.477, −0.362	
Direct effect	−0.386	0.030	−0.444, −0.328	
Total indirect effect	−0.034	0.009	−0.053, −0.017	8.11%

### 3.5 Moderated Mediation Model Testing

**FIGURE 3:**
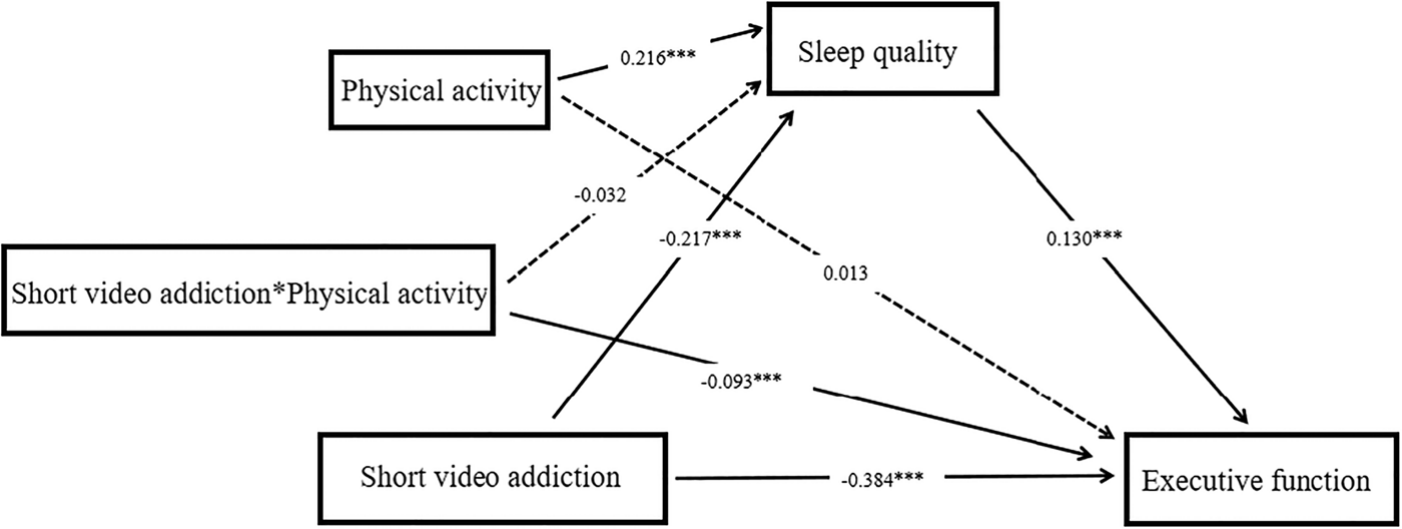
Mediating moderation model (*: p < 0.001).

After controlling for demographic variables, moderated mediation model analysis showed that SVA exerts a significant direct association with EF (β = −0.384, *p* < 0.001), alongside an indirect association mediated by SQ (β = −0.217, *p* < 0.001). PA was found to moderate the indirect path. Specifically, there was a significant negative correlation between SVA and SQ (β = −0.217, *p* < 0.001) and a significant positive correlation between SQ and EF (β = 0.130, *p* < 0.001). Additionally, the interaction term between PA and SVA showed a significant negative correlation with EF (β = −0.093, *p* < 0.001) (see **Table** **6**, Figure 3).

**TABLE 6:** Test of the moderated mediation effect model.

Outcome variables	Predictor variables	β	SE	*t*	*R* ^2^	*F*
SQ	SVA	−0.217	0.032	−6.825***	0.174	25.481***
	PA	0.126	0.033	3.783***		
	SVA*PA	−0.032	0.029	−1.105		
EF	SVA	−0.384	0.030	−12.826***	0.303	46.832***
	SQ	0.130	0.030	4.406***		
	PA	0.013	0.031	0.435		
	SVA*PA	−0.093	0.027	−3.509***		

***: p<0.001.

**FIGURE 4:**
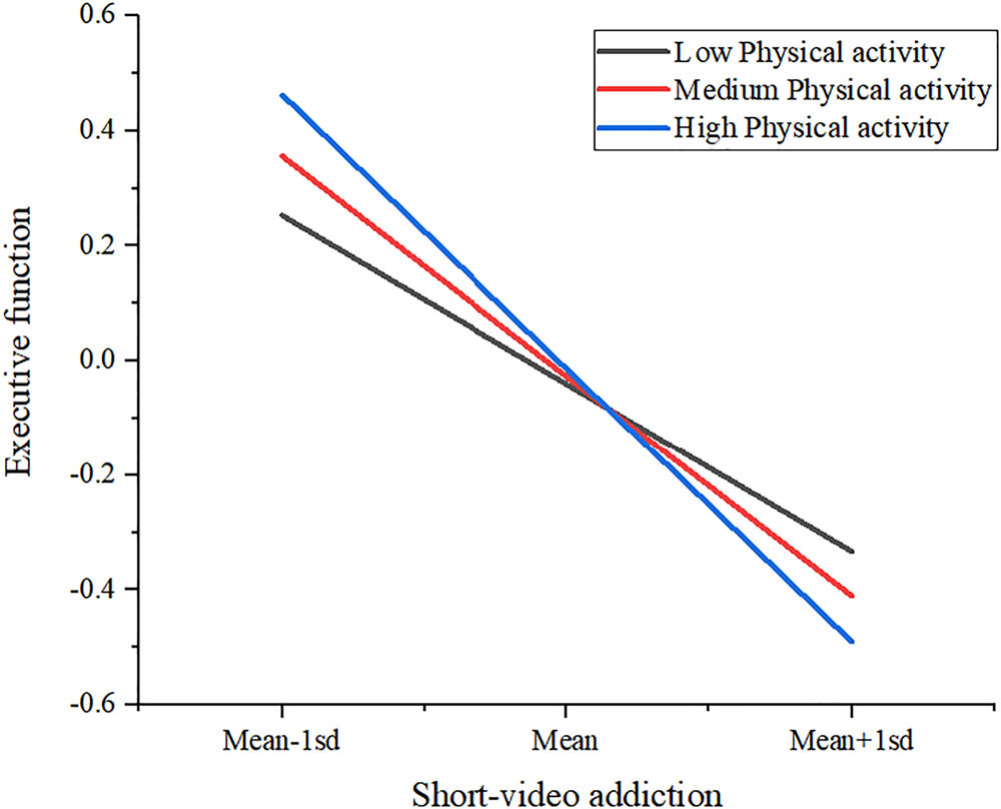
Simple slope graph.

Further simple slope analysis explored the relationship between SVA and EF at different levels of PA (low, moderate, and high). Specifically, a significant negative relationship was found between SVA and EF across all PA levels, and the strength of this relationship increased with the rise in PA level. At low PA, the negative correlation between SVA and EF was β = ‐0.293 (p < 0.001); at moderate PA, β = ‐0.384 (p < 0.001); and at high PA, β = ‐0.477 (p < 0.001). (See Table 7 and Figure 4)

**TABLE 7:** The moderating effects of different intensity physical activities on SVA and EF.

PA level	Effect size	SE	*t*	Lower limit 95% CI	Upper limit 95% CI
Low PA	−0.293	0.039	−7.569***	−0.369	−0.217
Medium PA	−0.384	0.030	−12.826***	−0.442	−0.325
High PA	−0.477	0.041	−11.715***	−0.557	−0.397

***: *p*<0.001.

## 4 Discussion

This study analyzes the relationships among SVA, EF, SQ, and PA in adolescents. Additionally, this study investigates the mediating role of SQ in the association between SVA and EF, along with the moderating role of PA in the relationships between SVA and EF, and between SVA and SQ. The results reveal significant negative correlations between SVA and SQ, PA, and EF. In contrast, SQ is positively correlated with both PA and EF, while a positive correlation also exists between PA and EF. After controlling for demographic variables, SQ partially mediates the relationship between SVA and EF in middle school students, and PA moderates the relationship between SVA and EF. These findings confirm our initial hypotheses.

The study confirmed a significant negative correlation between SVA and EF (H1 supported), a finding that is consistent with theoretical perspectives in the field of mobile phone addiction. Individuals with a high susceptibility to addiction exhibit notable deficits in inhibitory control, specifically in regulating impulsive behaviors (such as the inability to resist immediate gratification) and a decline in self‐management abilities (Hai 2015). The underlying neurophysiological mechanisms can be traced to dysfunction in the prefrontal cortex. According to the strength model of self‐control, sustained depletion of self‐regulation resources leads to compensatory deterioration in control abilities (Nilsen et al. 2020). This decline manifests at the level of EF in three typical symptoms: impulsive decision‐making tendencies (Cao et al. 2007; Salehi et al. 2022), risk‐taking behaviors (Seok et al. 2015), and working memory deficits. These dysfunctions are closely associated with abnormal activation in the dorsolateral prefrontal cortex (DLPFC) (Bari and Robbins 2013; Yuan and Raz 2014), and the neuroplasticity damage in the DLPFC caused by addictive behaviors may serve as the pathological basis for a “vicious cycle” of deteriorating EF and escalating addiction (Curtis and D'Esposito 2003).

The research revealed that SQ significantly serves as a partial mediator in the relationship between SVA and EF (H2 supported). The underlying mechanism can be decomposed into three pathways: First, sleep disturbances directly impair EF by inducing dysfunction in the prefrontal cortex (manifested as impaired abstract reasoning and goal‐directed behavior) (Mitru et al. 2002). Second, the degree of addiction to short videos is significantly correlated with SQ. The emission of blue light from electronic devices during the night has a negative impact on sleep regulation by inhibiting the production of melatonin (Sinha et al. 2022; Wahl et al. 2019). Furthermore, radiofrequency electromagnetic fields generated by electronic devices may disrupt metabolic processes and cerebral blood flow, consequently impairing sleep quality in adolescents (LIU et al. 2017). Poor SQ, in turn, leads to hindered memory consolidation (Walker et al. 2002), disrupting the cognitive resource integration required for EF. Third, chronic sleep deprivation induces daytime functional impairments (such as reduced alertness and insufficient cortical activation) (Dahl 1996), forming a vicious cycle of “addictive behavior—sleep deterioration—cognitive decline.” Neurophysiological evidence indicates that sleep durations below the 6.8‐hour threshold lead to systemic physiological disorders, including endocrine and metabolic dysfunctions (Wang and Bíró 2021). A meta‐analysis comprising 35 studies on obstructive sleep apnea (OSA) revealed that chronic sleep disturbances caused by OSA impair all domains of EF, with these impairments improving after treatment (Olaithe and Bucks 2013). These findings collectively suggest that improving SQ may be a crucial target for interrupting the neurotoxic transmission associated with SVA.

The analysis further identified a statistically significant negative correlation between SVA and SQ; however, PA did not significantly moderate this relationship (H3 unsupported). This result is inconsistent with previous studies on adolescent SVA (Wilckens et al. 2018), potentially due to the multi‐dimensional factors (such as nocturnal light exposure and cognitive arousal levels) that synergistically affect SQ. PA may exert long‐term effects through indirect pathways (such as stress regulation and circadian rhythm optimization) (Ashrafinia et al. 2014). Notably, PA exhibited a significant negative moderating effect on the relationship between SVA and EF (H4 supported), where higher activity levels corresponded to stronger detrimental effects of addictive behavior on EF. This phenomenon may arise from cognitive resource competition in high‐activity individuals, where academic stress and physical load together contribute to a reduction in prefrontal cortex compensatory function. The learning/development mechanism explains the relationship between PA and EF, suggesting that PA involves a learning process (Sibley and Etnier 2003). PA not only directly promotes changes in brain structure and function but also indirectly enhances EF by improving motivation, self‐efficacy, and social interaction abilities (de Bruijn et al. 2018). A meta‐analysis indicated that PA exerts a beneficial effect on cognitive performance in adolescents, with both short‐ and long‐term participation in PA improving EF, attention, and academic performance (de Greeff et al. 2018).

### 4.1 Innovation and Limitations

The novelty of this research primarily resides in its comprehensive analysis of the interactions among adolescent SVA, EF, SQ, and PA. Specifically, it investigates the mediating and regulatory functions of SQ and PA in the association between SVA and EF. The findings demonstrate a significant detrimental association between SVA and EF, while underscoring the contributory roles of SQ and PA within this dynamic. These outcomes yield novel perspectives on how SVA influences adolescent mental health and lay a theoretical groundwork for developing subsequent interventions. However, this study has several limitations. First, the reliance on self‐reported measures may introduce subjectivity, potentially influencing the accuracy and validity of the outcomes. Subsequent studies would benefit from integrating objective assessment tools to enhance data reliability. Additionally, this study employed a cross‐sectional design, limiting its ability to make causal inferences, direct interpretations, or genralizations of the findings. Therefore, to enhance the causal inference between variables, future studies would benefit from employing longitudinal designs. Moreover, the geographical concentration of the sample may limit the generalizability of the findings. Expanding recruitment to incorporate more diverse demographic and regional backgrounds would improve external validity and broaden the applicability of the results. Despite these constraints, the outcomes emphasize the potential benefits of enhancing sleep quality and promoting physical activity in mitigating short‐form video addiction among adolescents. This study offers novel insights and suggests promising avenues for further investigation. Subsequent research should examine the interplay between short‐form video addiction and other psychological health conditions, as well as the influence of cognitive biases, to further elucidate the complex mechanisms associated with SVA. Future research can adopt a longitudinal study design to track the long‐term causal relationships among SVA, EF, SQ, and PA and clarify the direction of influence among variables. In addition, future studies can expand the sample scope to include adolescents from more regions and ethnic groups to improve the generalizability of the research results. It is also necessary to explore the potential moderating or mediating effects of other variables, such as family parenting styles, peer relationships, and academic pressure, on the relationship between SVA and EF. Furthermore, combining objective measurement methods (e.g., actigraphy to assess sleep quality, fMRI to detect brain function changes) with subjective scales can provide more comprehensive and accurate research evidence. Finally, based on the research results, targeted intervention programs can be developed to help adolescents reduce short‐video addiction, improve sleep quality and physical activity levels, and thus promote the healthy development of their executive functions.

## 5 Conclusion

This study examined the association between SVA and adolescent EF and identified a significant negative correlation between the two. SQ partially mediated this relationship, and PA moderated the indirect pathway through which SVA links to EF via SQ. Moreover, the negative correlation between SVA and EF remained significant across all levels of PA. These findings underscore the importance of improving SQ and increasing PA as interventions to mitigate SVA and promote the development of EF in adolescents. Future research should further explore other potential influencing factors to better understand the complex mechanisms underlying the relationship between SVA and EF.

## Author Contributions

**Ting Xiao**: Conceptualization; Methodology; Data curation; Writing – Original Draft; Writing – Review & Editing. **Haiou Peng**: Methodology; Data curation; Writing – Review & Editing. **Zhiru Liang**: Data curation; Writing – Review & Editing; Funding acquisition. **Mengting Pang**: Conceptualization; Methodology; Data curation; Writing – Review & Editing. **Yang Liu**: Conceptualization; Methodology; Data curation; Writing – Original Draft; Writing – Review & Editing. **Yanfei Wang**: Data curation; Writing – Review & Editing; Funding acquisition. **Wenxi Tang**: Methodology; Data curation; Writing – Review & Editing; Funding acquisition. **Bo Wang**: Data curation; Writing – Review & Editing.

## Funding

The authors have nothing to report.

## Conflicts of Interest

The authors declare no conflicts of interest.

## Data Availability

The datasets generated and/or analysed during the current study are not publicly available due [our experimental team's policy] but are available from the corresponding author on reasonable request.
