# Correction to “When Screens Replace Sleep: Short Video Addiction Impairs Executive Function Through Sleep Disruption in Youth—Is Physical Activity the Antidote?”

**DOI:** 10.1002/brb3.71499

**Published:** 2026-05-25

Xiao, T., H. Peng, Z. Liang, et al. 2026. “When Screens Replace Sleep: Short Video Addiction Impairs Executive Function Through Sleep Disruption in Youth—Is Physical Activity the Antidote?.” *Brain and Behavior* 16, no. 4: e71369. https://doi.org/10.1002/brb3.71369

In the authors’ list, the order of the authors Bo Wang and Wenxi Tang has been interchanged.

The correct authors’ list is as follows:

Ting Xiao, Haiou Peng, Zhiru Liang, Mengting Pan, Yang Liu, Yanfei Wang, Bo Wang, Wenxi Tang.

The online version of this article has been corrected accordingly.

We apologize for this error.

